# Visual Impairment in the South Indian State of Andhra Pradesh: Andhra Pradesh - Rapid Assessment of Visual Impairment (AP-RAVI) Project

**DOI:** 10.1371/journal.pone.0070120

**Published:** 2013-07-23

**Authors:** Srinivas Marmamula, Saggam Narsaiah, Konegari Shekhar, Rohit C. Khanna, Gullapalli N. Rao

**Affiliations:** 1 Allen Foster Community Eye Health Research Centre, International Centre for Advancement of Rural Eye Care, L V Prasad Eye Institute, Hyderabad, India; 2 Bausch and Lomb School of Optometry, L V Prasad Eye Institute, Hyderabad, India; 3 Dana Center for Preventive Ophthalmology, Wilmer Eye Institute, Johns Hopkins School of Medicine, Baltimore, Maryland, United States of America; Medical University Graz, Austria

## Abstract

**Purpose:**

To assess the prevalence and causes of visual impairment in urban and rural population aged ≥40 years in the South India state of Andhra Pradesh.

**Methods:**

A population based cross-sectional study was conducted in which 7800 subjects were sampled from two rural and an urban locations. Visual Acuity (VA) was assessed using a tumbling E chart and eye examinations were performed by trained vision technicians. A questionnaire was used to collect personal and demographic information and previous consultation to eye care providers. Blindness and moderate Visual Impairment (VI) was defined as presenting VA <6/60 and <6/18 to 6/60 in the better eye respectively. VI included blindness and moderate VI.

**Results:**

Of the 7800 subjects enumerated, 7378 (94.6%) were examined. Among those examined, 46.4% were male and 61.8% of them had no education. The mean age of those examined (51.7 years; standard deviation 10.9 years) was similar to those not examined (52.8 years; standard deviation 9.9 years) (p = 0.048). Age and gender adjusted prevalence of VI was 14.3% (95% CI: 13.5–15.0). Refractive errors were the leading cause of VI accounting for 47.6% of all VI followed by cataract (43.7%). Together, they contributed to over 91.3% of the total VI. With multiple logistic regression, the odds of having VI increased significantly with increasing age. Those respondents who had no education were twice (95% CI: 1.7–2.5) more likely to have VI compared to those who were educated. VI was associated with rural residence (OR: 1.3; 95% CI: 1.1–1.6). The association between VI and gender was not statistically significant.

**Conclusions:**

The visual impairment remains a public health challenge in Andhra Pradesh, most of which can be addressed with relatively straight forward interventions like cataract surgery and spectacles. The eye care services need to be streamlined to address this challenge.

## Introduction

Sound epidemiological data is essential for developing strategies for blindness prevention. The last one and half decades have witnessed the emergence of rapid assessment surveys in eye care as a cornerstone for the planning and monitoring of eye care services in developing countries. [1], [2] With a massive global effort of eliminating avoidable blindness under the VISION 2020 : The Right to Sight initiative, the regular surveys have become more relevant than ever to assess the trends in prevalence of visual impairment and to assist in planning and monitoring of blindness prevention programmes worldwide. The recent figures indicate a decrease in the global burden of VI despite a demographic shift, which can be considered an important outcome of the intensive blindness prevention initiatives worldwide [3].

Andhra Pradesh is the one the largest states in India having a population of over 86 million as per the 2011 census, with over one third of the population being urban. [4] The Andhra Pradesh Eye Disease Study (APEDS) was the largest study that was undertaken in this state during 1996–2000 which revealed a blindness prevalence of 1.84% [5] and moderate visual impairment of 8.1% across all age groups. [5], [6], [7] A follow-up of the surviving cohort APEDS is underway.

We undertook a large cross sectional study to assess the prevalence and causes of visual impairment among those aged 40 years and older in two rural and an urban location in the south Indian state of Andhra Pradesh. Using a similar methodology, we earlier reported a 4.6% blindness and 9.4% moderate VI in weaving communities in Prakasam district in the same state in those aged 40 years and older [8].

## Methodology

### Study Area

**Figure 1:**
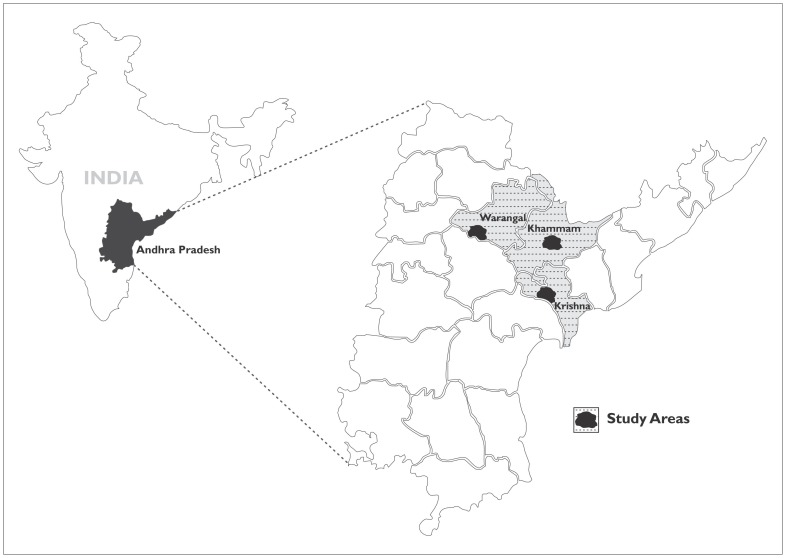
Map showing the three study locations; Warangal (rural), Khammam (rural) and Krishna (Vijayawada-urban).

The three study locations were Vijayawada (urban) in Krishna district, Khammam (rural) and Warangal (rural) (Figure 1) and the corresponding populations of these districts were 4,529,009, 2,798,241 and 3,522,644 respectively. [4] Vijayawada is the third largest city in Andhra Pradesh. It is a business centre and has several eye hospitals providing eye care services. The areas (sub-districts which comprise of group of villages) selected in Khammam district were Aswapuram, Burgampahad, Paloncha, Kothagudem, Tekulapalle and Mulkalapalle and in Warangal district, the sub-districts selected were Bachannapet, Narmetta, Station Ghanpur, Raghunathpalle, Jangaon and Lingala ghanpur. All the sub-districts selected in both the districts were predominantly rural but are reasonably well connected to the district headquarters. These sub-districts were within a radius of 50 kilometers from an eye care facility (secondary level eye hospital that is equipped to provide comprehensive eye care services including cataract surgery) associated with L V Prasad Eye Institute. This was done mainly for the provision of referral services for those who needed them and for the logistic support for the study.

### Ethics Approval

The study protocol was reviewed and approved by Institutional Review board (IRB) (Scientific and Ethics committee) of Hyderabad Eye Research Foundation, L V Prasad Eye Institute, Hyderabad, India. The study was conducted in accordance with the tenets of the Declaration of Helsinki. Permission was obtained from the head of the each village before starting the data collection. At household level, the study procedures were explained to each individual and Oral consent was obtained in presence of other family members and another individual who does not belong to same family, usually a neighbor. Each individual was free to decide on participation in the study. As the study used a simple and non invasive eye screening protocol, IRB granted permission for verbal consent. The studies were carried out in phases during year 2011 and 2012.

### Study Sample

The sample size was calculated with an expected prevalence of blindness of 6%, precision 20% with 95% error bound, and 10% non response rate. The sample size is calculated to be approximately 2600 from each of the three study areas. A multi-stage sampling procedure was used to select the study sample. A total of 52 clusters are randomly selected from each of the three study districts. In each cluster, the area was demarcated, mapped and segmented in such a way that each segment contained the required number of households to provide at least 50 individuals aged 40 years and older. One of the segments was randomly selected for the study. Villages and municipals wards were used as clusters in rural and urban area respectively.

The timing of the survey was planned in such a way that the maximum number of participants was available in their homes for examination. These included visits in the morning and during weekends, especially in the urban area. All the individuals in each of the selected household fulfilling the age criteria were listed and all those available were examined. At least two attempts were made to examine those who were not available at the first visit. Those who were still not available after repeated attempts were considered as not available and were not substituted so that bias in recruitment could be minimized.

### Eye Examination

The subjects were visited in their households and eye examination was performed by one of the three teams each comprising of a vision technician (personnel trained for one year to provide primary eye care in rural areas) and community eye health worker. The vision technicians received special training in the survey procedures and documentation of findings. A reliability study was set-up prior to the main study and all three vision technicians had good agreement on visual acuity measurements (kappa statistic 0.7 or greater) with the gold standard senior optometrist.

Distance Visual acuity (VA) was assessed using a standard Snellen chart with tumbling E optotypes at distance of 6 meters, outdoors, and in shade on bright and sunny days. Due precautions were taken to avoid reflections and glare on the chart. If a subject was unable to identify letters on the first line of the chart, then the distance between the chart and the subject was reduced to 3 meters and then to 1 meter respectively and VA recording was attempted. Unaided VA was recorded on all subjects. Aided VA was recorded if a subject reported the use of spectacles. Among those who had no spectacles, unaided VA was considered as presenting VA and among those who had spectacles, aided VA was considered as presenting VA. If presenting VA was less than 6/12, the VA was recorded using a multiple pinhole occulder. Near vision was assessed binocularly using the N notation chart at a fixed distance of 40 cm for each individual. Fixed distance was maintained by using a string attached to the near vision chart. Both unaided and aided near vision were assessed if the subject reported using spectacles. Near vision was re-assessed among subjects who had near vision <N8 by using near addition lenses in a trial frame appropriate for that age. External examination was performed using torchlight. Lens was assessed using distant direct ophthalmoscopy and the lens were graded as Normal, Obvious lens opacity, Aphakia or Pseudophakia. If the lens could not be examined due to conditions like corneal opacities, phthisis or absent globe, then it was documented.

A brief interview was done which collected personal and demographic information, spectacle use, use of eye drops and previous consultation for any eye problems including details of any surgery in the past. If the subjects had undergone any previous consultation, the reports were asked for and details of treatment taken were documented in the survey record. If a subject had visual impairment for distance or near, then the reasons for not utilizing the eye care services was asked and documented.

### Definitions

We used Indian definitions for categories of visual impairment (VI). According to this, blindness is defined as presenting VA less than 6/60 in the better eye. This included the blindness and severe visual impairment categories as defined by World Health Organization. Moderate VI was defined as presenting VA less than 6/18 to 6/60. The Indian definitions were used to facilitate comparisons with previous studies from India. [9], [10] Cataract is defined as opacity of crystalline lens obscuring the red reflex partially or completely on distance direct ophthalmoscopy and causing visual impairment. Refractive error was deemed to be present if presenting distance VA was worse than 6/18 and improving to 6/18 or better with a pinhole. The principle of visual impairment was recorded for each eye separately and then for the person. If there was more than one cause, the one that was more easily treatable or correctable was marked as the main cause of visual impairment. All the subjects identified with visual impairment were referred to the secondary eye care facility for management.

### Data Management

Data were initially collected on RAVI data collection forms and entered into a database created in Microsoft access. Regular consistency checks were performed. Data analysis was performed using SPSS 16.0 (SPSS Inc., Chicago, IL). Estimated prevalence was adjusted to the age and gender distribution of population of Andhra Pradesh estimated for the year 2011. Prevalence with 95% confidence intervals (CI) are presented. The demographic associations of visual impairment with age, gender, education, area of residence were assessed using multivariate analysis using multiple logistic regression models and adjusted odds ratios (OR) with 95% CI are reported. The fitness of the regression model was assessed using Hosmer-Lemeshow test for goodness of fit.

## Results

Of the 7800 subjects enumerated, 7378 (94.6%) were examined. The participation rates were 94.4% in Vijayawada, 95.6% in Khammam and 93.8% in Warangal. Among those examined 46.4% were male and 61.8% of them had no education. The mean age of those examined (51.7 years; standard deviation 10.9 years) was similar to those not examined (52.8 years; standard deviation 9.9 years) (p = 0.048). Women were more likely to be examined compared to men (95.6% versus 93.5%; p<0.01).

### Prevalence of Visual Impairment

A total of 918 subjects had VI. After adjusting for age and gender distribution of the population of 2011, the prevalence of VI was 14.3% (95% CI: 13.5–15.0). Table 1 shows the prevalence of VI in all the three study locations along with causes of VI. It ranged from 10.2% (95% CI: 9.0–11.4) in Vijayawada (urban) to 14.2% (95% CI: 12.67–15.47) in Warangal (rural).

**Table 1:** Prevalence and causes of visual impairment in three studies areas.

	Vijayawada (%)	Khammam (%)	Warangal (%)	All areas combined (%)	% of total visual Impairment
Refractive Error	4.73	6.88	6.15	6.62	**47.60**
Cataract	4.28	4.95	7.10	6.32	**43.68**
Posterior segment disorder	0.49	0.32	0.41	0.47	**3.27**
Uncorrected Aphakia	0.53	0.56	0.12	0.52	**3.27**
Surgery related complications	0.08	0.00	0.21	0.12	**0.76**
Corneal scar	0.08	0.12	0.08	0.10	**0.76**
Phthisis bulbi	0.00	0.12	0.12	0.10	**0.65**
**Total**	**10.18 (9.01–11.44)**	**12.96 (11.66–14.34)**	**14.19 (12.67–15.47)**	**14.25 (13.45–15.05)**	**100.0**

Values are expressed as percentage prevalence.

* Visual impairment (VI) is defined as presenting visual acuity <6/18 in the better eye.

† Prevalence adjusted to the age and gender distribution of the population of Andhra Pradesh in year 2011.

Overall, the age and gender adjusted prevalence of blindness in all the areas combined was 5.5% (95% CI: 5.0–6.0). It ranged from 3.6% (95% CI: 3.0–4.6) in Vijayawada to 5.6% (95% CI: 4.7–6.6) in Warangal. Similarly, the prevalence of moderate VI ranged from 6.4 (95% CI: 5.5–7.5) to 8.6 (95% CI: 7.5–9.8) in Vijayawada and Warangal respectively. Both blindness and moderate VI were significantly higher in rural areas compared to urban location (p<0.05, Chi squared test) (Table 2).

**Table 2:** Categories of visual impairment based on presenting visual acuity in the better eye stratified by area of residence.

	Moderate Visual Impairment (Presenting Visual Acuity <6/18–6/60 in the better eye)	Blindness (Presenting Visual Acuity <6/60 in the better eye)
	Prevalence (%) (95% confidence intervals)	Prevalence (%) (95% confidence intervals)
**All study participants**		
**Urban**		
Vijayawada (n = 2455)	6.4 (5.5–7.5)	3.6 (3.0–4.6)
**Rural**		
Khammam (n = 2485)	8.4 (7.3–9.5)	4.6 (3.8–5.5)
Warangal (n = 2438)	8.6 (7.5–9.8)	5.6 (4.7–6.6)
**Combined rural (n = 4923)**	8.5 (7.7–9.3)	5.10 (4.5–5.8)
**All areas combined (n = 7378)** †	8.8 (8.2–5.5)	5.5 (5.0–6.0)
**Among ≥50 years and older (sub-sample)**		
**Urban**		
Vijayawada (n = 1178)	12.2 (10.3–14.1)	7.3 (5.8–8.8)
**Rural**		
Khammam (n = 1191)	17.1 (5.8–8.8)	9.2 (7.6–10.8)
Warangal (n = 1357)	12.5 (10.7–14.3)	9.7 (8.1–11.3)
**Combined rural (n = 2548)**	14.7 (13.3–16.1)	9.5 (8.4–10.6)
**All areas combined (n = 3726)** †	14.1 (12.9–15.3)	9.0 (8.0–10.0)

* Proportions are presented with 95% confidence intervals in parenthesis.

† Prevalence adjusted for age and gender distribution of the population of Andhra Pradesh in year 2011.

Among the subset of the sample, aged 50 years and older, the age and gender adjusted prevalence of VI was 23.1% (95% CI: 21.8–24.5) and included 9.0% (95% CI: 8.0–9.9) of blindness and 14.1% (95% CI: 12.9–15.3) moderate VI impairment. Both blindness and visual impairment were higher in the rural area compared with the urban location (Table 2).

With multiple logistic regression, the odds of having VI increased significantly with increasing age. Compared to the 40–49 year old age group, the odds of having VI among those aged 50–59, 60–69 and 70 years and older were 6.0 (95% CI :4.5–8.0), 18.5 (95% CI: 14.0–24.5) and 46.7 (95% CI: 34.8–62.7) respectively. Those respondents who had no education were twice (95% CI: 1.7–2.5) more likely to have VI compared to those who were educated. VI was associated with rural residence (OR: 1.3; 95% CI: 1.1–1.6). The association between VI and gender was not statistically significant (OR: 1.1; 95% CI: 0.9–1.3, p = 0.3) (Table 3).

**Table 3:** Multivariate analysis showing the association between visual impairment and socio-demographic variables.

	Total in the group	No. of Visually impaired‡	Adjusted Odds Ratio (95% Confidence interval)
	(n = 7378)	(n = 918)	
	n	n (%)	
**Age group (years)** *			
40–49	3652	63 (1.7)	1.0
50–59	1774	184 (10.4)	6.0 (4.5–8.0)
60–69	1277	342 (26.8)	18.5 (14.0–24.5)
70 & above	675	329 (48.7)	46.7 (34.8–62.7)
**Gender** †			
Male	3421	404 (11.8)	1.0
Female	3957	514 (13.0)	1.1 (0.9–1.3)
**Education level** *			
Any education	2816	159 (5.7)	1.0
No Education	4562	759 (16.6)	2.0 (1.7–2.5)
**Area** *			
Urban	2455	250 (10.2)	1.0
Rural	4923	668 (13.6)	1.3 (1.1–1.6)

* p<0.001;

† p = 0.30;

‡ Visual impairment (VI) is defined as presenting visual acuity <6/18 in the better eye.

### Causes of Visual Impairment

**Figure 2:**
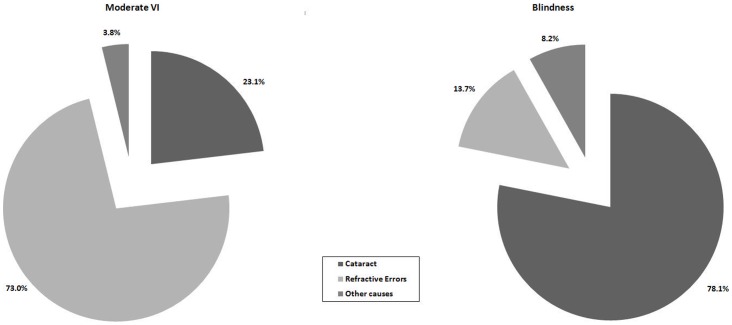
Pie-chart showing the causes of visual impairment among those who had moderate visual impairment and blindness.

Refractive errors were the leading cause of VI accounting for 47.6% of all VI followed by cataract (43.7%). Together, they contributed to over 91.3% of the total VI. The cataract and refractive errors were the leading cause of blindness and moderate VI respectively (Figure 2). In younger individuals (40–69 years) refractive errors was the leading cause of VI whereas cataract was the leading cause in older individuals (70 years and older). A comparison of causes of visual impairment among males and females, levels of education and area of residence are presented in Table 4.

**Table 4:** Main causes of visual impairment in the study population and demographic characteristics.

		Causes of Visual impairment (%)		
	Total visually impaired	Cataract	Refractive error†	Other causes
	(n = 918)	(n = 401)	(n = 467)	(n = 50)
	n	(%)‡	(%)‡	(%)‡
**Age group (years)**				
40–49	63	28.6	58.7	12.7
50–59	184	41.3	57.1	1.6
60–69	342	41.2	55.3	3.5
70 & above	329	50.5	41.3	8.2
**Gender**				
Male	404	44.6	49.8	5.7
Female	514	43.0	51.8	5.3
**Education level**				
Any education	159	38.4	54.7	6.9
No Education	759	44.8	50.1	5.1
**Area**				
Urban	250	42.0	51.6	6.4
Rural	668	44.3	50.6	5.1

† includes people with uncorrected aphakia.

‡ Row percentages presented.

## Discussion

We reported prevalence and causes of VI in the south Indian state of Andhra Pradesh. The cross sectional nature of the study with high response rate and similar urban and rural distribution of our sample to that of the state are the strengths of our study. Cataract was the cause for 44% of the total blindness and 40% of the total VI in the Andhra Pradesh Eye Disease Study compared to 78.1% and 23.1% in the present study. [5] This difference could be due to variation in study methodology, age groups and case definitions used and variations in assigning the cause of visual impairment. We found both the moderate VI and blindness were higher than that reported from cloth weaving communities [8] but lower than that reported from Fishing communities in Andhra Pradesh using a similar methodology. [11] However, both of the studies were conducted in specific population groups whereas the present was done in general population that limit cross comparisons. The prevalence of moderate VI and blindness in the present study and previous studies conducted in the state of Andhra Pradesh are compared in Table 5. It indicates that both moderate VI and blindness continue to be prevalent in Andhra Pradesh and that there is significant regional variation within the State of Andhra Pradesh.

**Table 5:** Results from previous population based studies conducted in Andhra Pradesh, India.

Place of survey	Age Group studied (years)	Year of study	Sample size (examined)	Blindness (presenting visual acuity <6/60 in the better eye	Moderate visual impairment (presenting visual acuity <6/18 to 6/60 in the better eye)
				Prevalence (%) (95% Confidence intervals)	Prevalence (%) (95% Confidence intervals)
Hyderabad, West Godavari, Adilabad, Mahabubnagar districts [5], [6]	All ages	1996	10293	1.84% (1.5–2.2)	8.1% (7.5–9.3)
Adilabad district (RACSS) [27]	≥50	2006–2007	2160	8% (6.9–9.1)	13.6% (12.2–15.1)
Adilabad district (APEDS) [27]	≥50	1998–1999	521	11% (8.3–13.7)	40.3% (20.9–49.3)
Prakasam district (Cloth weaving communities) [8]	≥40	2011	2848	4.6% (3.8–5.5)	9.4% (8.3–10.5)
Prakasam district (Fishing communities) [11]	≥40	2010	1560	7.1% (5.8–8.4)	22.7% (20.6–24.8)
Present Study (Krishna, Khammam, Warangal districts)	≥40	2011–2012	7378	5.5% (5.0–6.0)	8.8% (8.2–8.8)

The national survey in India showed a prevalence of blindness and moderate visual impairment as 8% and 16.8% respectively [12] similar to 9.0% and 14.0% respectively in the present study in the same age groups (50 years and older). Similar to our findings, cataract was the leading cause of visual impairment. [12] Despite the continuous efforts by several stakeholders to increase availability and accessibility of services as indicated by increasing cataract surgical coverage (number of cataract surgeries performed per million population per year), cataract remains a major public health problem in India with regional variations similar to other developing countries in Asia. [12], [13], [14], [15], [16]. As suggested by Murthy et. al, strong monitoring mechanism and population based surveillance systems are required to prioritize the regions where additional efforts and resources are required. [17].

Consistent with other studies in India and the rest of the world, we observed a significant increase in the odds of VI in elderly population. [5], [12], [13], [13], [14], [16,18] An aging population can offset the efforts of prevention of blindness programmes unless appropriate measures are taken to account for the increasing demand for services. [19].

Refractive errors, though recently recognized as a problem of public health, are the leading cause of VI. About two-thirds of moderate VI and 14% of blindness is attributed to refractive errors in our study. Considering its importance, refractive errors are one of the five priorities under VISION 2020 initiative; several service delivery models are being implemented cross the world to address the issue of uncorrected refractive errors. [20] One such model, the ‘village vision complex’ with vision centre as a primary eye care unit is being planned in two of the three study districts. [21], [22], [23].

The association between female gender and VI is found to differ across the studies. While the study from Gujarat in India [24], China [25] and Latin America [26] found no association, other studies from India found a significant association between gender and VI. [5], [12] We found an increase in the odds of VI among women but it was not statistically significant. It is possible that some unknown socio-demographic factor is influencing this trend.

Consistent with other studies in India, we found a higher prevalence of VI in rural areas compared to urban areas. [5], [12] Though prevalence of VI in Warangal was higher compared Khammam, the difference was not statistically significant. As expected, the VI was lower in Vijayawada which was an urban location. Possible reasons could be the difference in availability, accessibility, awareness and affordability of services in rural and urban areas. We found a higher prevalence of VI among those who are not educated as reported earlier. [5], [18], [24], [25] It can be speculated that education is linked with higher socio-economic status, increased awareness on availability of care and uptake of services resulting in a lower prevalence of visual impairment.

The most recent population based studies were conducted in older individuals aged 50 and older though a few studies are reported among 40 and younger age groups. As VI is more prevalent in older age groups, restricting the study to the older age groups will result in smaller sample size, logistically more feasible and thus less expensive. However, inclusion of individuals aged 40 years and older will provide an opportunity to collect additional information on presbyopia with little extra cost. Though our sample included individuals aged 40 years and older, we also reported prevalence estimates for 50 year old age groups to facilitate comparison with similar studies.

While high response rates and representative nature of our sample are the strengths, the study is not immune to limitations. As we used a rapid assessment methodology, there is a possibility of overestimation of cataract diagnosis by virtue of the examination protocol used. Assessment of posterior segment disease through undilated pupil is a challenge in field settings and might have been responsible for underestimating its prevalence as a cause of visual impairment.

A comprehensive eye care service delivery (village vision complex with a secondary eye care centre and complementary vision centres to provide primary eye care) is being developed in Khammam and Krishna district in Andhra Pradesh in India and the present study is designed to provide the baseline data for this initiative. [23] Only a repeat of this survey after 5–7 years could show the impact of the service delivery model in this region, through there is an evidence for this from other locations in the state. [23], [27] The Warangal district where no intervention is planned may act as a control for future comparisons.

In summary, visual impairment remains a public health challenge in Andhra Pradesh most of which can be addressed with relatively straight forward interventions like cataract surgery and spectacles. Unless the comprehensive eye care strategies reach the remote rural locations, the goal of elimination of avoidable blindness by year 2020 will remain a dream to be accomplished in Andhra Pradesh, India.
